# Employing nanoparticle tracking analysis of salivary neuronal exosomes for early detection of neurodegenerative diseases

**DOI:** 10.1186/s40035-023-00339-z

**Published:** 2023-02-07

**Authors:** Vaibhav Sharma, Fredrik Nikolajeff, Saroj Kumar

**Affiliations:** 1 Department of Health, Education and Technology, Lulea University of Technology, Lulea, Sweden; 2 Department of Biophysics, All India Institute of Medical Sciences, New Delhi, India

**Keywords:** Exosomes, Parkinson’s disease, Alzheimer’s disease, Neurodegenerative disease, Diagnosis, Prognosis, Extracellular vesicles, Nanoparticle tracking analysis

## Abstract

Neurodegenerative diseases are a set of progressive and currently incurable diseases that are primarily caused by neuron degeneration. Neurodegenerative diseases often lead to cognitive impairment and dyskinesias. It is now well recognized that molecular events precede the onset of clinical symptoms by years. Over the past decade, intensive research attempts have been aimed at the early diagnosis of these diseases. Recently, exosomes have been shown to play a pivotal role in the occurrence and progression of many diseases including cancer and neurodegenerative diseases. Additionally, because exosomes can cross the blood–brain barrier, they may serve as a diagnostic tool for neural dysfunction. In this review, we detail the mechanisms and current challenges of these diseases, briefly review the role of exosomes in the progression of neurodegenerative diseases, and propose a novel strategy based on salivary neuronal exosomes and nanoparticle tracking analysis that could be employed for screening the early onset of neurodegenerative diseases.

## Background

Timely diagnosis of neurodegenerative diseases at an early stage provides an effective means to manage the ‘impending burden’ of the aging population globally. Alzheimer’s disease (AD) and Parkinson’s disease (PD) are the most common neurodegenerative diseases. In terms of prevalence, AD tops the list, followed by PD. The other spectrum of neurodegenerative diseases encompasses frontotemporal dementia (FTD), Huntington’s disease (HD), and amyotrophic lateral sclerosis (ALS) [1, 2]. Accumulation of amyloid fibrils in tissues is linked to many neurodegenerative diseases, although the associated protein varies among diseases, for instance, amyloid β (Aβ) in AD and α-synuclein (α-syn) in PD [3]. However, there is increasing evidence for significant overlap between misfolding proteins and various symptoms in these diseases [4]. AD is clinically characterized by decline in cognitive abilities and changes in behavior whereas pathologically, by extracellular and intracellular deposits of insoluble Aβ and neurofibrillary tangles (NFTs), respectively [5]. These senile amyloid plaques result from either overproduction of Aβ (~ 1% in familial forms) or dysfunction of Aβ clearance, which is hypothesized to be the main cause of Aβ accumulation in sporadic AD that accounts for 99% of total AD cases [6, 7]. On the contrary, NFTs consist of abnormally hyperphosphorylated insoluble forms of Tau (τ) protein. Both Aβ accumulation and NFTs can exert direct and indirect neurotoxic effects leading to inflammation that causes neuronal death. Hence, Aβ amyloid, total tau, and phosphorylated tau are considered “core” AD biomarkers [8].

On the other hand, PD is a chronic neurodegenerative disease characterized by motor impairments due to the death of dopaminergic neurons within the substantia nigra. Cognitive impairments can also follow during the course of the disease [9]. Generally, familial forms that are sporadic in origin but rare, are linked to mutations in *SNCA*, *parkin*, *DJ-1, PINK-1* (PTEN-induced kinase 1), and Leucine-rich repeat kinase 2 (*LRRK2*) [10]. Although the detailed molecular pathogenesis is still unclear, it is widely accepted that α-syn plays a central role in causing PD pathology and is in turn responsible for neurodegeneration [11–13].

Both of these neurodegenerative diseases are currently incurable, and the pathological events start a decade before symptoms become severe enough to be diagnosed clinically using the current criteria. The development of PD has three stages, preclinical, prodromal, and clinical, which are characterized by the onset of neurodegeneration in the absence of symptoms, the presence of non-motor symptoms, and the occurrence of motor symptoms, respectively [14, 15]. The diagnosis of PD occurs at the clinical stage based on the presence of bradykinesia along with either tremor or rigidity. It is worth noting that by this time 70%–80% of dopamine has been lost in the striatum [16]. Similarly, to improve AD diagnosis, the progression of the disease is broken down majorly into three categories: preclinical, mild cognitive impairment (MCI), and dementia [17, 18]. The preclinical stage signifies no cognitive symptoms but some signs of pathology upon brain imaging, whereas MCI represents a transition zone between normal aging and AD. It is noteworthy that the annual conversion rate of MCI to AD is around 30% [19, 20]. Finally, accurate and early diagnosis is further complicated by overlaps in clinical features that hinder the discrimination between diseases. For example, mild PD associated with dementia has similar patterns of cognitive dysfunction as mild AD.

ALS is a neuromuscular disorder resulting from protein inclusions formed from TAR DNA-binding protein of 43 kDa (TDP-43), Cu/Zn superoxide dismutase (SOD-1) or fused in sarcoma (FUS) within motor neurons [21], which result in loss of motor functions, progressive degeneration and ultimately death from asphyxiation or inanition [22]. Sporadic ALS accounts for almost 90% of the cases and the remaining 10% falls under the familial form of the disease [23]. The diagnosis of ALS is difficult due to the variability in patient presentation and lack of a definitive biomarker for the disease. A primary pathological marker of ALS is the deposition of ubiquitinylated or hyperphosphorylated protein inclusions in motor neurons and glia of the spinal cord/brainstem and motor cortex. Most of the cases (~ 97%) show TDP-43 pathology rather than SOD-1 (~ 2%) or FUS (~ 1%) aggregates. It is also worth noting that aggregates of SOD-1 or FUS are only associated with mutations, whereas TDP-43 aggregates can result from the wild-type protein in a sporadic form and be associated with mutations in familial ALS [24].

HD is also an incurable neurodegenerative disease and the most common form of inherited neurodegenerative disease. HD is characterized by uncontrolled and excessive motor movements along with a significant cognitive loss. HD is associated with abnormal expansion of a CAG repeat in the *IT15* gene that results in abnormal numbers of glutamine repeats (polyQ) in the huntingtin (Htt) protein. Six to 34 CAG repeats are normal but a person with over 40 repeats will develop HD and usually die 10–15 years after disease onset [25]. Therefore, a pathological hallmark of HD is the intracellular aggregates of mutant Htt called inclusion bodies. The most accepted causes for aggregation of mutant Htt are “proteasomal impairment” and various posttranslational modifications such as ubiquitination, sumoylation and acetylation [26, 27].

**Fig. 1:**
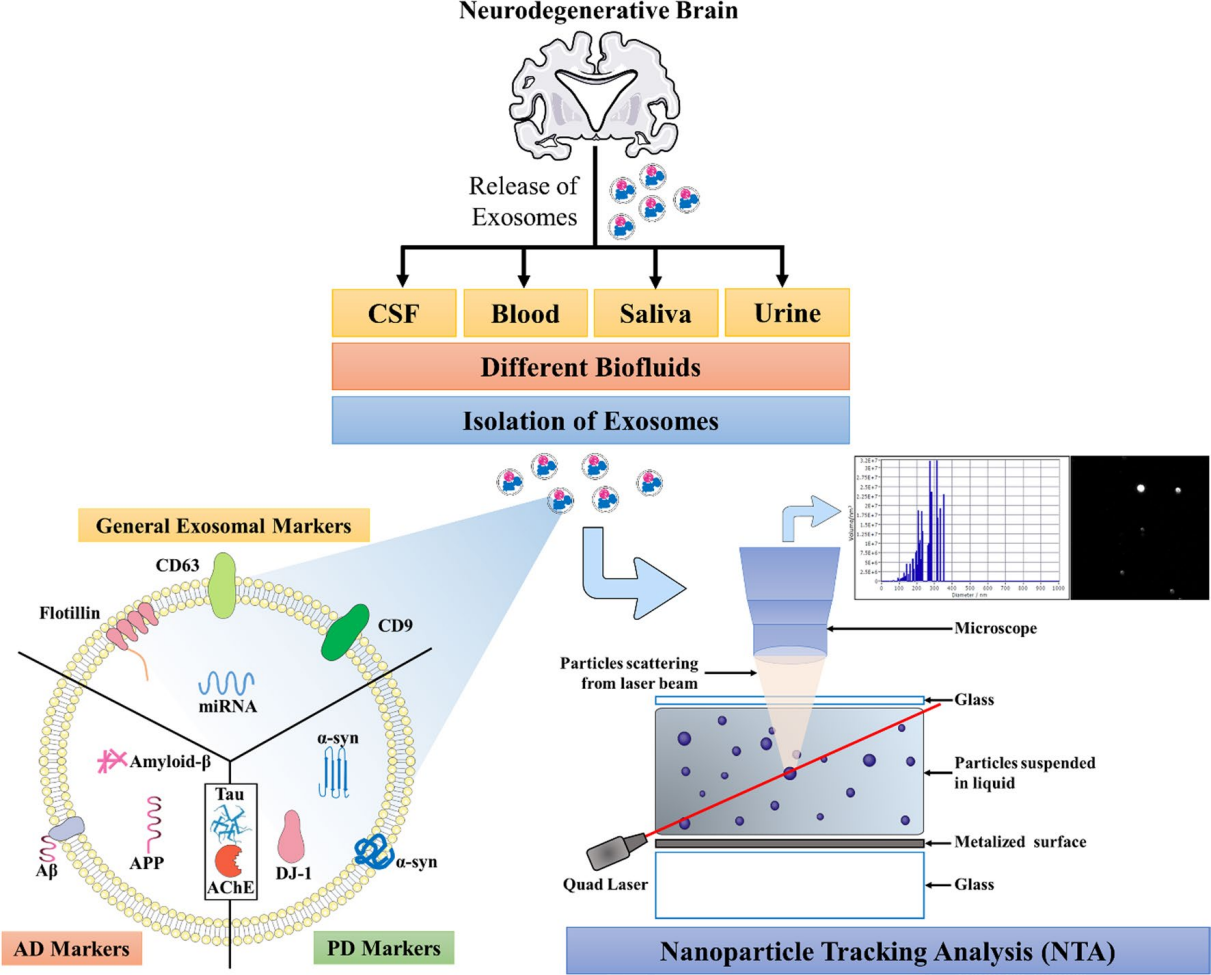
Schematic representation of general characteristics of exosomes and their characterization via NTA. Differing surface markers in the context of different neurodegenerative diseases are also presented

With a need for an efficient diagnostic methodology for neurodegenerative diseases, a variety of approaches have been tried ranging from tracking subtle functional changes in the brain using positron emission tomography (PET) [28–30] to identifying biomarkers in the skin [9] and body fluids like blood [31–33], urine [34, 35], cerebrospinal fluid [36–38], and whole saliva [39–41]. The utility of volatile organic compounds in breath samples has also been explored for early diagnosis and staging of PD [42]. Recently, exosomes from brain cells are considered as a fundamental mediator of intercellular communication that can dynamically reflect the pathological state of the donor cells. Despite their involvement in age-related neurodegeneration, research on exosomes and their cargos in neurodegenerative diseases is still in its infancy [43] (Fig. 1).

### Exosomes and their role in neurodegenerative diseases

**Fig. 2:**
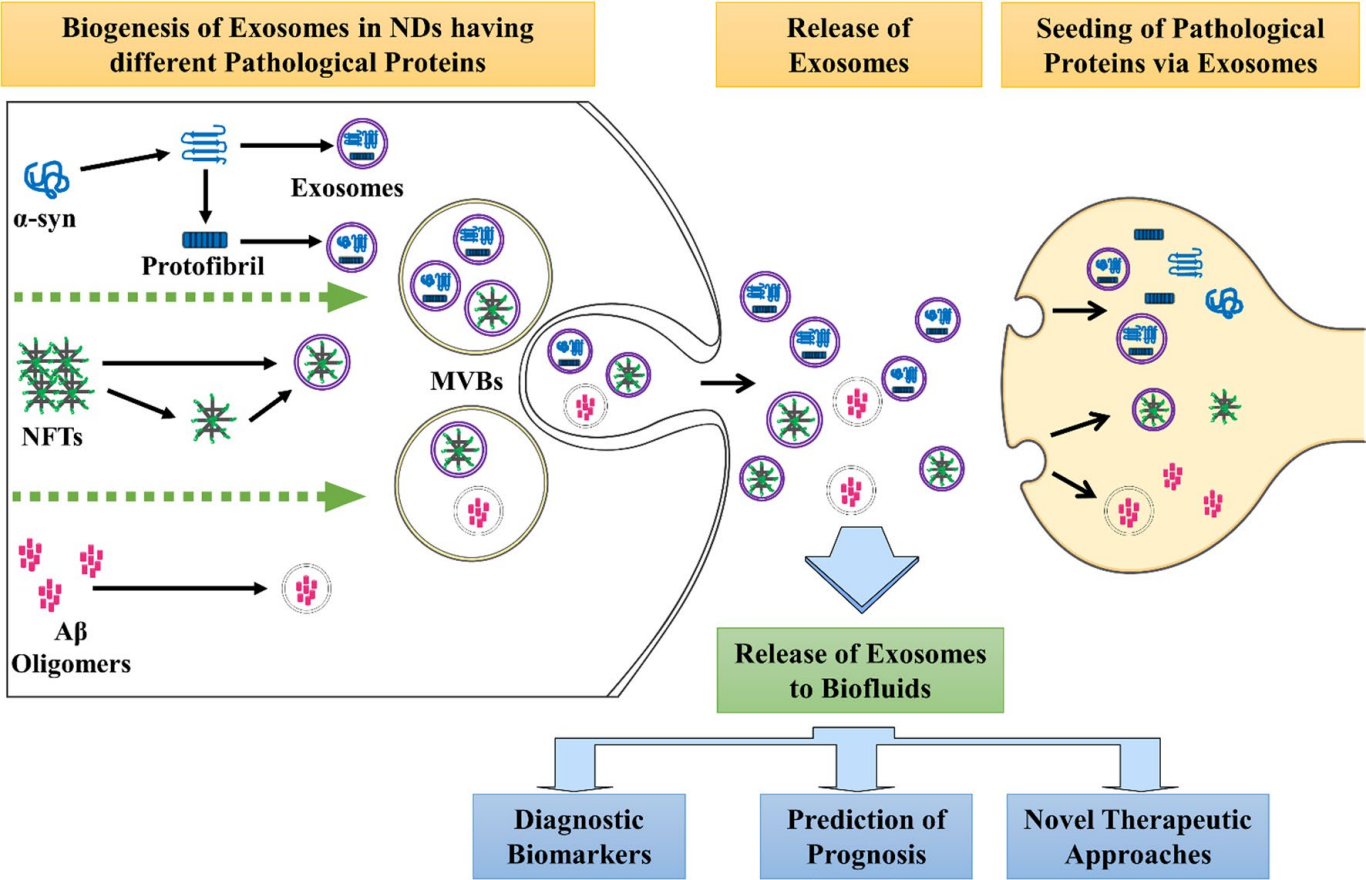
Exosome biogenesis in neuronal cells and their role in cell-to-cell transmission of various “infectious proteins”. These “cargo-loaded vesicles” are also released in the circulatory system. α-syn: alpha-synuclein; NFT: neurofibrillary tangle; Aβ: amyloid beta; MVB: multivesicular body

Exosomes are tiny extracellular vesicles ranging from 30 to 100 nm in diameter, and are released by all cell types. These vesicles are formed from late endosomes via inward budding of multivesicular bodies (MVBs). Invagination of endosomal membranes results in the formation of intraluminal vesicles (ILVs) within large MVBs (Fig. 2). During this process, certain proteins may be trapped into the invaginating membrane, and cytosolic materials are also engulfed and enclosed into the ILVs. The majority of ILVs that are released into extracellular space via fusion with the plasma membrane are termed “exosomes”. A small proportion of MVBs function as “garbage trucks” by guiding their contents to lysosomes for degradation and removal. RAB7 and RAB2 are primary mediators that coordinate traffic between late endosomes and lysosomes. RAB GTPases are a family of small GTPases belonging to the RAS superfamily [44]. Also, MVBs sometimes do not fuse directly to lysosomes but via autophagosome-dependent fusion to form auto-phagolysosomes, which degrade the content they encapsulate. However, the detailed mechanism that sorts MVB to plasma membrane and lysosome is unclear, but there exists a decision point between the two fates, i.e., inhibition of one path will increase the other [45, 46]. Alternatively, the formation of ILVs requires the function of endosomal sorting complex required for transport (ESCRT), a complex protein machinery consisting of four separate ESCRTs (ESCRT-0, -I, -II, and -III) that cooperate to facilitate MVB formation, protein cargo sorting and vesicle budding [47–49]. Initially, exosomes were considered as a mere waste-disposing system. It is now widely accepted that they play a central role in intercellular communication [50, 51]. Exosomes serve as a vehicle for not only proteins but also DNA, mRNA, miRNA, and other non-coding RNAs (ncRNAs) and thus contribute to the modulation of gene expression within target cells. In addition, specific markers on the membrane of exosomes reflect their origin as well as differentiating them from other extracellular vesicles. CD63, CD81, CD9, ALIX, and TSG101 are generally considered specific markers for exosomes [52, 53]. Neural-derived exosomes can largely be detected by the presence of L1-cell adhesion molecule (L1CAM), which is a central nervous system-specific marker [54, 55]. However, quite recently, there have been some contradicting reports questioning the use of L1CAM as a neuronal specific marker [56] as it is shown to be present in other cell types like T and B cells and at higher levels in several cancer types [57].

Extracellular vesicles including exosomes can be isolated by several techniques such as differential ultracentrifugation, size exclusion chromatography, ultrafiltration, immunoaffinity capture, and polyethylene glycol-based precipitation. Efficient isolation of exosomes from different biofluids has been largely studied and nicely reviewed elsewhere [58–62], thus will not be discussed in detail here. Each of these isolation methods has its pros and cons as discussed in the cited studies above, but it is worth noting that despite recent technological advances in this field, it is still difficult to obtain pure and homogeneous exosomal preparations in sufficient quantities by any of the currently available methods or technologies. In recent years, several studies have compared performances of different methods and their combinations in the isolation of exosomes from various biofluids [63–66], and shown that exosome isolation from the same biomaterial by different methods may vary significantly in the yield, purity, and biochemical compositions of exosomes. The type of biological fluid can also affect the parameters. Furthermore, the exosomal pool itself is quite heterogenous [67], consisting of subpopulations that differ in size, morphology, surface markers, and biochemical compositions [68].

Another important class of molecules contained within exosomes are miRNAs, which are usually small, non-coding, and about 22 nucleotides in length. A miRNA can repress the translation or regulate the degradation of over 100 mRNAs and one mRNA may be regulated by multiple miRNAs. Thus, they form a powerful gene regulation network and are involved in key biological processes including cell signaling, apoptosis, neuronal development, and plasticity [69, 70]. miRNAs are enriched in exosomes compared to cell-free serum or plasma because they are relatively more stable within exosomes [71, 72]. These exosomal miRNAs may be useful diagnostic and prognostic biomarkers of diseases. Besides being highly variable across neurodegenerative diseases, exosomal miRNAs can be used to discriminate disease subtypes [73]. For instance, the clinical phenotype of multiple sclerosis can be accurately distinguished via expression of different miRNAs at different stages. Ebrahim khani et al. identified 9 miRNAs that can differentiate relapsing-remitting multiple sclerosis from secondary/primary progressive multiple sclerosis [74].

Furthermore, exosomes have been strongly linked to the pathogenesis and progression of many neurodegenerative diseases [75, 76]. In the case of PD, the exosomal biogenesis machinery has been implicated in α-syn accumulation [77], and both in vitro and in vivo studies have noted pathological propagation of Tau aggregates by exosomes [78, 79]. It has also been reported that exosomes provide an ideal environment for α-syn to aggregate and this exosomal form of oligomeric α-syn is more easily taken up by recipient cells compared with the free form, resulting in potential propagation of the PD pathology [80, 81] (Fig. 2).

Some exosomal miRNAs have also been reported as PD biomarkers. For instance, miR-19b, miR-24 and miR-195 can be used for PD diagnosis, based on the target scan tool. Cao et al. [82] related these miRNAs to pathological process in PD, such as miR-19b related to Parkin RBR E3 ubiquitin protein ligase, miR-19b to LRRK2/PARK8, as well as miR-24 and miR-195 to ATP13A2/PARK9. Another study reported a panel of 5 exosomal miRNAs from CSF, comprising Let-7f-5p, miR-27a-3p, miR-125a-5p, miR-151a-3p and miR-423-5p, which shows 90% sensitivity and 80% specificity for differentiation of PD from controls [83].

Another set of misfolded proteins including superoxide dismutase 1 and TDP-43, which have shown associations with ALS, have been identified in exosomes [84, 85]. Also, in the case of AD, exosomes extracted from the brains of AD patients show significantly elevated levels of amyloid precursor protein, Aβ oligomers, Aβ_1−42_, and p-S396-tau [86–88]. Additionally, significant differences in miR-9-5p and miR-598 are detected in exosomes from CSF of AD  vs control participants [89]. Another study has shown that the serum exosomal miR-135a and miR-384 are upregulated in AD patients when compared with healthy cohorts, and miR-384 could be used for discriminating between AD, vascular dementia and Parkinson’s disease with dementia [90].

Several studies have pointed out that exosomes can also transport the expanded polyglutamine tract of both Htt RNA and protein as well as the mutant huntingtin protein (mHtt) aggregates, and thus trigger HD-related behavioral and pathological features [90–92]. Htt is a large protein of 350 kDa, which makes it hard to be packed into exosomes. Therefore, mHtt packaging and spreading through exosomes, which is supported by recent evidence [93], is a complex pathway that remains to be defined.

FTD is considered as a second most common cause of dementia with an age of onset < 65. In FTD, the most common mutations are from three genes: granulin (*GRN*) [94], *C9orf72* (chromosome 9 open reading frame 72) [95] and *MAPT* (microtubule associated tau) [96]. It has been documented that many proteins involved in FTD pathogenesis are secreted by cells in association with exosomes. Furthermore, mutations in *GRN* result in the reduction of exosomes and the alteration of their composition [97]. Neurofilament light chain has been considered as a biomarker for axonal injury and is reportedly increased in sera of FTD patients [98, 99]. Another report showed higher levels of exosomal heat shock protein-70 (HSP70) than free HSP70 in plasma in FTD and AD, and the exosomal HSP70 level correlated with ^18^F-FDG-PET [100].

Recently, the biomarker potential of salivary neuronal exosomes has been explored in neurodegenerative diseases. Saliva is a type of easily accessible biofluid and isolation of salivary exosomes is a non-invasive, painless, and relatively simple procedure when compared with blood and CSF sampling [101]. Some recent studies have shown increased levels of α-syn oligomers within salivary exosomes form PD patinets when compared with the healthy cohorts [102, 103]. The only reported study connecting salivary exosomes concentration to cognitive impairment (CI) and AD showed an increased concentration of salivary exosomes in CI and AD than in healthy controls. The αβ oligomer and p-tau show high protein abundance in salivary exosomes in CI and AD in comparison to control subjects [104]. Despite the limited number of studies on salivary neuronal exosomes to date, the saliva-derived exosomes hold promise for various clinical applications, including use in a non-invasive biomarker panel and for disease progression tracking [105].

On the other hand, some research findings indicate that the production of exosomes may be involved in the improvement of pathological phenotype of diseases like AD [106, 107] and PD [108, 109]. In summary, exosomes can be considered as a “double-edged sword” suggesting their neuropathologic and neuroprotective roles. On the one hand, exosomes are involved in the dysregulation of communication between neurons or between neurons and glial cells, which triggers neurodegenerative diseases. On the other side, there is evidence for the involvement of exosomes in sequestering neurotoxic molecules from neural cells and the transfer of neuroprotective ones [110, 111]. However, the detailed mechanisms of the switch between the two sides remain unknown.

## Nanoparticle tracking analysis (NTA): an emerging platform for screening neurodegenerative diseases

**Fig. 3:**
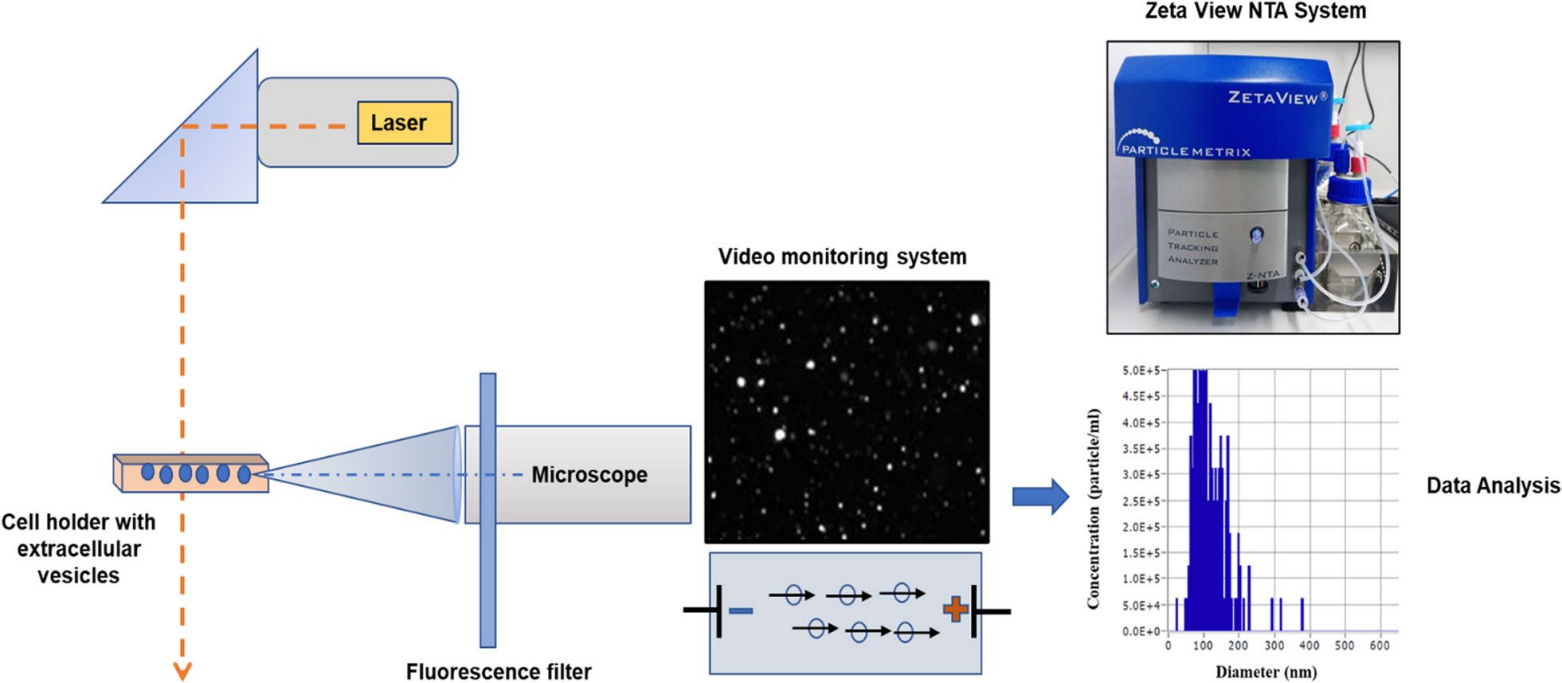
Schematic setup of a nanoparticle tracking analyzer. The particles in a sample are illuminated with a laser beam. The scattered light is recorded by a CMOS camera fitting at 90˚ to the illumination plane, in a built-in ultramicroscope system. A fluorescence filter is placed between the cell holder and the microscope. Light scattered by the particles is displayed in the “live-view” window of the software. The size of each particle is calculated by Brownian motion analysis of the individual tracks, allowing for simultaneous determination of size and concentration

NTA has emerged as a state-of-the-art method for the characterization of exosomes [112, 113]. This method combines two different physical principles, light scattering and Brownian motion (Fig. 3). First, particles in liquid suspension are irradiated by a laser beam and the 2D trajectory of each particle is tracked to calculate the diffusion coefficient. Second, a video of the displacement of each particle under Brownian motion is captured by camera (Fig. 3). The hydrodynamic radius for individual particles is calculated via the Stokes–Einstein equations [114, 115] as follows:

$$\overline{{\left( {x,y} \right)^{2} }} = \frac{{2kTt}}{{3R_{h} \eta }}$$

where *k* = Boltzmann constant, *T =* temperature, and *η* = solvent viscosity. The particle size is reported as the hydrodynamic radius, *R*_h_, determined by following the 2D trajectory of each particle over a tracking time *t*.

The advantage of NTA is that individual particles are tracked, hence one can obtain both the concentration and the size distribution, rather than just a mean size. One disadvantage with NTA is that smaller particles are not easily measurable, as the minimum size for detection depends on the refractive index increment. In addition, particles larger than 1 μm diffuse slowly and thus are also difficult to measure with NTA [116]. A seemingly similar instrument called differential light scattering (DLS) also works on the scattering principle and measures particle diffusion. However, the DLS obtains the total contribution from all scattering particles, which is slightly biased towards larger-sized particles and thereby swamps out contributions from smaller particles, while in NTA, more accurate measurements can be obtained by its particle-by-particle approach [117]. Many initial studies with NTA were focused on the validation of NTA measurements of mono- and multi-modal nanoparticles and compared its performance with DLS [114, 118]. When analyzing polydisperse samples such as proteins and vesicles, NTA provides a much better resolution than DLS [119, 120].

The aggregation of proteins is central to many neurodegenerative disorders. The NTA system has been used for the characterization of fibrillar protein aggregates including Aβ and polyglutamine peptides [121], and analysis of aggregation kinetics of α-syn [122]. The NTA system can also be used to monitor bioconjugation. For instance, gold nanoparticle binding to protein A and subsequent interactions with immunoglobulin G can be measured with NTA, using hydrodynamic radius as a function of measurement [123, 124]. A more recent study has shown the utility of statistical mixture distribution combined with NTA to quantitate the amount and extent of particle binding in a mixture of nanomaterials [125]. Furthermore, antibody- and fluorescence-based NTA methods have been used to study specific populations of exosomes and provide better insights. Both antibody- and fluorescence-based methods allow accurate sizing and phenotyping of various exosomes based on their respective surface markers [126–128]. With the NTA system, many studies have reported an increase of neuronally derived exosomes in various biofluids in neurodegenerative disorders. For instance, in PD, brain-derived exosomes in plasma were found to be significantly increased when compared with the age-matched healthy controls [129]. In another supportive study, increased DJ-1 and α-syn were found in neural-derived exosomes from PD patients [130]. In the saliva samples from PD [131, 132] and AD patients [133], neuronal salivary exosomes were found to be increased in a similar pattern. This approach could be extended to other neurodegenerative diseases as well [62]. Hence, combining the specificity of fluorescence and antibody-based exosomal quantifications through the NTA system could serve as a promising screening methodology for a variety of neurodegenerative diseases, depending on their respective surface markers.

## Conclusion

Despite significant advances in clinical imaging technologies, there exist several unaddressed challenges in the accurate diagnosis of neurodegenerative diseases. As the molecular changes in these diseases begin as early as 10–20 years prior to the clinical manifestations, tracking molecular events could aid in the early diagnosis. In this regard, exosomes are considered as new “hotspots” since their role in the progression of various diseases is turning out to be quite critical. Through this article, we put forward a screening technology, which could be employed along with other already established subjective assessments and imaging methods for early diagnosis of neurodegenerative disorders. Fluorescence NTA has the potential to quantitate neuronally derived exosomes from diseased and healthy cohorts, based on their specific surface markers.

As of now, there are several studies elucidating concentration differences of exosomal particles between diseased and healthy volunteers. But cautiously, more studies with increased sample sizes are needed to validate this methodology and  make it a “mass-screening technology” in the future. Another necessity of particular interest is the follow-up of participants in such studies to gain more confidence in such a screening methodology. In the foreseeable future, more efforts should be made in similar areas to tackle the increasing burden of neurodegenerative diseases, in addition to drug-based efforts for curing these debilitating diseases.

## Data Availability

Not applicable.

## Abbreviations

NDs: Neurodegerative diseases
AD: Alzheimer’s disease
PD: Parkinson’s disease
FTD: Frontotemporal dementia
HD: Huntington’s disease
ALS: Amyotrophic lateral sclerosis
Aβ: Amyloid β
α-syn: α-Synuclein
NFT: Neurofibrillary tangle
LRRK2: Leucine-rich repeat kinase 2
MCI: Mild cognitive impairment
PET: Positron emission tomography
L1CAM: L1-cell adhesion molecule
TDP-43: Transactive response DNA binding protein 43 kDa
NTA: Nanoparticle tracking analysis
DLS: Differential light scattering
FUS: Fused in sarcoma
ESCRT: Endosomal sorting complex required for transport
ILV: Intraluminal vesicle
